# Supplementary material for: Novel Pharmacological Treatment Options in Pediatric Glioblastoma—A Systematic Review
Source: Cancers (Basel). 2022 Jun 6;14(11):2814. doi: 10.3390/cancers14112814 (PMC9179254; doi:10.3390/cancers14112814)
Supplement: Supplementary file 1 [file cancers-14-02814-s001.zip › Table S1.pdf]

cancers

MDPI

Supplementary Data:

Novel Pharmacological Treatment Options in Pediatric Glioblastoma—A Systematic Review

Johanna Wyss [1,2,*,†], Nicole Alexandra Frank [3,†], Jehuda Soleman [3,4,5,‡] and Katrin Scheinemann [1,6,7,‡]

Table S1. Predefined parameters according to PICO for evidence-based medicine.

Population	Intervention	Comparison	Outcome
Pediatric patients with GBM; age 0–18 years	Pharmacological treatment other than monotherapy with TMZ	No comparison (NA)	OS; PFS; EFS

GBM = glioblastoma, TMZ = temozolomide, NA = not applicable, OS = overall survival, PFS = progression free survival, EFS = event free survival.
